# Supplementary material for: Conditional chemoconnectomics (cCCTomics) as a strategy for efficient and conditional targeting of chemical transmission
Source: eLife. 2024 Apr 30;12:RP91927. doi: 10.7554/eLife.91927 (PMC11060718; doi:10.7554/eLife.91927)
Supplement: Figure 3—source data 3. [file elife-91927-fig3-data3.zip › Figure 3 source data3 part 2.zip!/Figure 3-Source data 3-part2/1060f2-1-CRISPResso_Report_gl5umwyq.zip!/CRISPResso_on_1060f2_1/2b.Nucleotide_percentage_quilt_around_sgRNA_gcccacttcctggagtatcg.pdf]

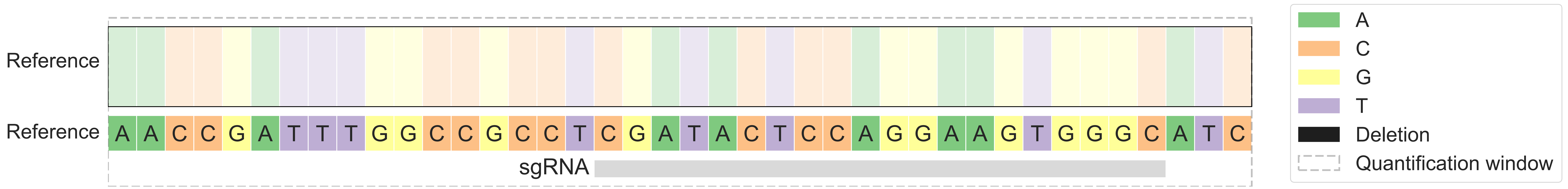
Reference
Reference
A A C C G A T T T G G C C G C C T C G A T A C T C C A G G A A G T G G G C A T C
sgRNA
A
C
G
T
Deletion
Quantification window
